# Bis(4-meth­oxy-3,4-di­hydro­quinazolin-1-ium) chloranilate

**DOI:** 10.1107/S1600536813023635

**Published:** 2013-08-31

**Authors:** Kazuma Gotoh, Hiroyuki Ishida

**Affiliations:** a Department of Chemistry, Faculty of Science, Okayama University, Okayama 700-8530, Japan

## Abstract

In the title compound [systematic name: bis­(4-meth­oxy-3,4-di­hydro­quinazolin-1-ium) 2,5-di­chloro-3,6-dioxo­cyclo­hexa-1,4-diene-1,4-diolate], 2C_9_H_11_N_2_O^+^·C_6_Cl_2_O_4_^2−^, the chloranil­ate anion lies about an inversion center. The 4-meth­oxy-3,4-di­hydro­quinazolin-1-ium cations are linked on both sides of the anion *via* bifurcated N—H⋯(O,O) and weak C—H⋯O hydrogen bonds, giving a centrosymmetric 2:1 aggregate. The 2:1 aggregates are linked by another N—H⋯O hydrogen bond into a tape running along [1-10]. The tapes are further linked by a C—H⋯O hydrogen bond into a layer parallel to the *ab* plane.

## Related literature

For NMR and nuclear quadrupole resonance (NQR) studies on proton-transfer in the short hydrogen-bond of the diazine–chloranilic acid (2:1) system, see: Nihei *et al.* (2000 ▶); Seliger *et al.* (2009 ▶). For a related structure, see: Gotoh & Ishida (2011 ▶). For the double π system of the chloranilate anion, see: Andersen (1967 ▶); Benchekroun & Savariault (1995 ▶).
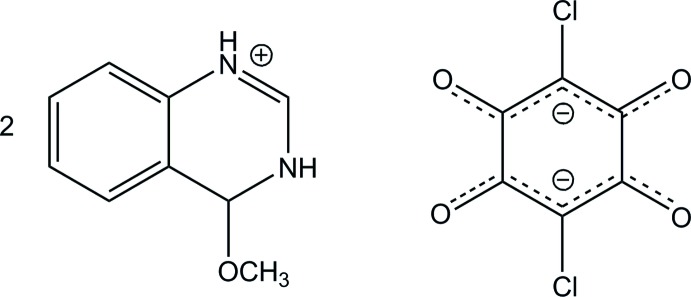


## Experimental

### 

#### Crystal data

2C_9_H_11_N_2_O^+^·C_6_Cl_2_O_4_^2−^
*M* *_r_* = 533.37
Triclinic, 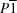

*a* = 4.9971 (4) Å
*b* = 8.6363 (4) Å
*c* = 13.5808 (9) Å
α = 97.869 (4)°
β = 91.660 (6)°
γ = 100.968 (5)°
*V* = 569.06 (7) Å^3^
*Z* = 1
Mo *K*α radiation
μ = 0.34 mm^−1^
*T* = 200 K
0.45 × 0.35 × 0.04 mm

#### Data collection

Rigaku R-AXIS RAPID II diffractometer
Absorption correction: numerical (*NUMABS*; Higashi, 1999 ▶) *T*_min_ = 0.887, *T*_max_ = 0.987
7422 measured reflections
2692 independent reflections
1913 reflections with *I* > 2σ(*I*)
*R*_int_ = 0.199

#### Refinement

*R*[*F* ^2^ > 2σ(*F* ^2^)] = 0.082
*wR*(*F* ^2^) = 0.177
*S* = 0.84
2692 reflections
172 parameters
H atoms treated by a mixture of independent and constrained refinement
Δρ_max_ = 0.89 e Å^−3^
Δρ_min_ = −0.54 e Å^−3^

### 

Data collection: *PROCESS-AUTO* (Rigaku/MSC, 2004 ▶); cell refinement: *PROCESS-AUTO*; data reduction: *CrystalStructure* (Rigaku/MSC, 2004 ▶); program(s) used to solve structure: *SHELXS97* (Sheldrick, 2008 ▶); program(s) used to refine structure: *SHELXL97* (Sheldrick, 2008 ▶); molecular graphics: *ORTEP-3 for Windows* (Farrugia, 2012 ▶); software used to prepare material for publication: *CrystalStructure* and *PLATON* (Spek, 2009 ▶).

## Supplementary Material

Crystal structure: contains datablock(s) General, I. DOI: 10.1107/S1600536813023635/hg5342sup1.cif

Structure factors: contains datablock(s) I. DOI: 10.1107/S1600536813023635/hg5342Isup2.hkl

Supplementary material file. DOI: 10.1107/S1600536813023635/hg5342Isup3.cml

Additional supplementary materials:  crystallographic information; 3D view; checkCIF report

## Figures and Tables

**Table 1:** Hydrogen-bond geometry (Å, °)

*D*—H⋯*A*	*D*—H	H⋯*A*	*D*⋯*A*	*D*—H⋯*A*
N1—H1⋯O1	0.95 (3)	1.79 (3)	2.706 (2)	160 (3)
N1—H1⋯O2^i^	0.95 (3)	2.56 (3)	3.229 (3)	127 (2)
N2—H2⋯O2^ii^	0.90 (3)	1.87 (3)	2.762 (3)	171 (3)
C4—H4⋯O1^iii^	0.95	2.34	3.214 (3)	152
C10—H10⋯O1	0.95	2.52	3.230 (3)	131

Symmetry codes: (i) 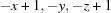; (ii) 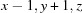; (iii) 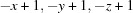.

**Fig. 1.**
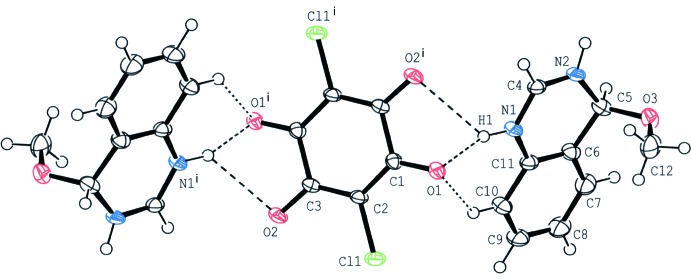
The molecular structure of the title compound, with the atom-labeling. Displacement ellipsoids of non-H atoms are drawn at the 50% probability level. The dashed lines indicate N—H···O and C—H···O hydrogen bonds. [Symmetry code: (i) -x + 1, -y, -z + 1.]

**Fig. 2.**
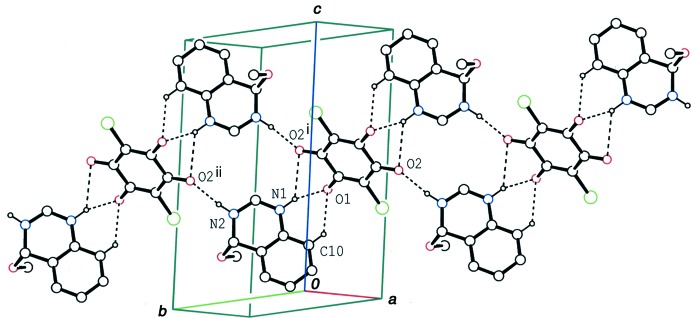
A partial packing diagram of the title compound. The dashed lines indicate N—H···O and C—H···O hydrogen bonds. H atoms of the not involved in the hydrogen bonds have been omitted. [Symmetry codes: (i) -x + 1, -y, -z + 1; (ii) x - 1, y + 1, z.]

## supplementary crystallographic information

### 1. Comment

The title compound was accidentally obtained in the preparation process of bis(quinazolinium) chloranilate, which is an interesting candidate for the study on proton-transfer in short hydrogen-bonded systems (Nihei *et al.*, 2000, Seliger *et al.*, 2009) and also for the study on *D*—H···*A* hydrogen bonding (*D* = N, O, or C; *A* = N, O or Cl) in amine–chloranilic acid systems (Gotoh & Ishida, 2011).

In the crystal structure of the title compound, an acid-base interaction involving proton transfer is observed between chloranilic acid and 4-methoxy-3,4-dihydroquinazoline. The chloranilate ion shows a characteristic structure having four short C—C bonds and two extremely long C—C bonds, and C—O with similar bond lengths, which is explainable in terms of the double π system of the anion (Andersen, 1967; Benchekroun & Savariault, 1995). One chloranilate anion and two 4-methoxy-3,4-dihydroquinazolin-1-ium cations are linked by bifurcated N—H···(O,O) and weak C—H···O hydrogen bonds (N1—H1···O1, N1—H1···O2^i^ and C10—H10···O1; symmetry code as in Table 1) to afford a centrosymmetric 2:1 aggregate (Fig. 1). The 2:1 aggregates are linked by another N—H···O hydrogen bond (N2—H2···O2^ii^; symmetry code as in Table 1), forming a tape along the [110] direction (Fig. 2). The tapes are further linked by a C—H···O hydrogen bond (C4—H4···O1^iii^; symmetry code as in Table 1) into a layer parallel to the *ab* plane.

### 2. Experimental

To a solution of chloranilic acid (183 mg) in acetonitrile (40 ml), a solution of quinazoline (228 mg) in acetonitrile (40 ml) was added at room temperature. A brown precipitate, which was immediately formed after mixing the solutions, was collected by filtration and then dissolved in methanol (40 ml). Single crystals of the title compound were obtained by slow evaporation from the methanol solution for *ca* three months at room temperature. During the slow evaporation process, quinazoline reacted with methanol under an acidic condition of chloranilic acid, yielding 4-methoxy-3,4-dihydroquinazoline.

### 3. Refinement

C-bound H atoms were positioned geometrically (C—H = 0.95 or 0.98 Å) and refined as riding, allowing for free rotation of the methyl group. *U*_iso_(H) values were set at 1.2*U*_eq_(C) or 1.5*U*_eq_(methyl C). The N-bound H atom was found in a difference Fourier map and refined isotropically. The refined N—H distances are 0.90 (3) and 0.95 (3) Å. The quality of the crystals studied were low as indicated by *R*_int_ = 0.199. This is possibly due to a small amount of quinazoline which remained without reacting with methanol and incorporated in the crystallization of the title compound as an impurity.

### Crystal data

**Table:**

2C_9_H_11_N_2_O^+^·C_6_Cl_2_O_4_^2^^−^	*Z* = 1
*M**_r_* = 533.37	*F*(000) = 276.00
Triclinic, *P*1	*D*_x_ = 1.556 Mg m^−^^3^
Hall symbol: -P 1	Mo *K*α radiation, λ = 0.71075 Å
*a* = 4.9971 (4) Å	Cell parameters from 6359 reflections
*b* = 8.6363 (4) Å	θ = 3.0–28.0°
*c* = 13.5808 (9) Å	µ = 0.34 mm^−^^1^
α = 97.869 (4)°	*T* = 200 K
β = 91.660 (6)°	Platelet, brown
γ = 100.968 (5)°	0.45 × 0.35 × 0.04 mm
*V* = 569.06 (7) Å^3^	

### Data collection

**Table:**

Rigaku R-AXIS RAPID II diffractometer	1913 reflections with *I* > 2σ(*I*)
Detector resolution: 10.00 pixels mm^-1^	*R*_int_ = 0.199
ω scans	θ_max_ = 27.9°
Absorption correction: numerical (*NUMABS*; Higashi, 1999)	*h* = −6→6
*T*_min_ = 0.887, *T*_max_ = 0.987	*k* = −11→11
7422 measured reflections	*l* = −17→17
2692 independent reflections	

### Refinement

**Table:**

Refinement on *F*^2^	Primary atom site location: structure-invariant direct methods
Least-squares matrix: full	Secondary atom site location: difference Fourier map
*R*[*F*^2^ > 2σ(*F*^2^)] = 0.082	Hydrogen site location: inferred from neighbouring sites
*wR*(*F*^2^) = 0.177	H atoms treated by a mixture of independent and constrained refinement
*S* = 0.84	*w* = 1/[σ^2^(*F*_o_^2^) + (0.*P*)^2^] where *P* = (*F*_o_^2^ + 2*F*_c_^2^)/3
2692 reflections	(Δ/σ)_max_ < 0.001
172 parameters	Δρ_max_ = 0.89 e Å^−^^3^
0 restraints	Δρ_min_ = −0.54 e Å^−^^3^

### Special details

**Table:**

Geometry. All e.s.d.'s (except the e.s.d. in the dihedral angle between two l.s. planes) are estimated using the full covariance matrix. The cell e.s.d.'s are taken into account individually in the estimation of e.s.d.'s in distances, angles and torsion angles; correlations between e.s.d.'s in cell parameters are only used when they are defined by crystal symmetry. An approximate (isotropic) treatment of cell e.s.d.'s is used for estimating e.s.d.'s involving l.s. planes.
Refinement. Refinement of *F*^2^ against ALL reflections. The weighted *R*-factor *wR* and goodness of fit *S* are based on *F*^2^, conventional *R*-factors *R* are based on *F*, with *F* set to zero for negative *F*^2^. The threshold expression of *F*^2^ > σ(*F*^2^) is used only for calculating *R*-factors(gt) *etc*. and is not relevant to the choice of reflections for refinement. *R*-factors based on *F*^2^ are statistically about twice as large as those based on *F*, and *R*- factors based on ALL data will be even larger.

### Fractional atomic coordinates and isotropic or equivalent isotropic displacement parameters (Å^2^)

**Table:**

	*x*	*y*	*z*	*U*_iso_*/*U*_eq_	
Cl1	0.92850 (10)	−0.01581 (6)	0.33371 (4)	0.0289 (2)	
O1	0.6877 (3)	0.26227 (17)	0.41520 (12)	0.0253 (4)	
O2	0.6806 (3)	−0.27488 (17)	0.44963 (13)	0.0273 (4)	
O3	0.0538 (3)	0.67492 (19)	0.17432 (13)	0.0325 (4)	
N1	0.3013 (4)	0.3978 (2)	0.33662 (15)	0.0238 (4)	
N2	0.0001 (4)	0.5674 (2)	0.32482 (16)	0.0264 (4)	
C1	0.6067 (4)	0.1368 (2)	0.45169 (16)	0.0202 (5)	
C2	0.6949 (4)	−0.0070 (2)	0.42490 (16)	0.0216 (5)	
C3	0.6061 (4)	−0.1443 (2)	0.46922 (16)	0.0208 (5)	
C4	0.1661 (4)	0.5056 (2)	0.37648 (17)	0.0239 (5)	
H4	0.1907	0.5399	0.4462	0.029*	
C5	−0.0403 (4)	0.5353 (3)	0.21674 (18)	0.0276 (5)	
H5	−0.2413	0.5032	0.2003	0.033*	
C6	0.0872 (4)	0.3967 (3)	0.17605 (18)	0.0252 (5)	
C7	0.0414 (5)	0.3326 (3)	0.07619 (19)	0.0329 (5)	
H7	−0.0787	0.3723	0.0350	0.039*	
C8	0.1683 (6)	0.2118 (3)	0.0362 (2)	0.0375 (6)	
H8	0.1365	0.1689	−0.0323	0.045*	
C9	0.3429 (5)	0.1532 (3)	0.0965 (2)	0.0367 (6)	
H9	0.4315	0.0707	0.0688	0.044*	
C10	0.3890 (5)	0.2130 (3)	0.19591 (19)	0.0305 (5)	
H10	0.5075	0.1721	0.2370	0.037*	
C11	0.2585 (4)	0.3353 (3)	0.23554 (17)	0.0234 (5)	
C12	0.3396 (6)	0.7357 (3)	0.1882 (2)	0.0405 (6)	
H12A	0.3821	0.8406	0.1661	0.061*	
H12B	0.4377	0.6626	0.1493	0.061*	
H12C	0.3958	0.7459	0.2589	0.061*	
H1	0.413 (6)	0.351 (3)	0.377 (2)	0.037 (7)*	
H2	−0.097 (6)	0.628 (4)	0.362 (2)	0.043 (8)*	

### Atomic displacement parameters (Å^2^)

**Table:**

	*U*^11^	*U*^22^	*U*^33^	*U*^12^	*U*^13^	*U*^23^
Cl1	0.0312 (3)	0.0265 (4)	0.0301 (4)	0.0090 (2)	0.0144 (2)	0.0008 (3)
O1	0.0295 (8)	0.0181 (7)	0.0299 (9)	0.0075 (6)	0.0073 (6)	0.0041 (7)
O2	0.0324 (8)	0.0188 (8)	0.0327 (9)	0.0111 (6)	0.0113 (7)	0.0006 (7)
O3	0.0428 (10)	0.0251 (8)	0.0326 (10)	0.0131 (7)	0.0048 (7)	0.0057 (8)
N1	0.0288 (9)	0.0188 (9)	0.0246 (10)	0.0091 (7)	0.0052 (7)	−0.0013 (8)
N2	0.0314 (10)	0.0213 (9)	0.0281 (11)	0.0106 (8)	0.0125 (8)	0.0000 (9)
C1	0.0219 (9)	0.0162 (10)	0.0217 (11)	0.0032 (8)	0.0015 (8)	0.0005 (9)
C2	0.0234 (10)	0.0189 (10)	0.0223 (11)	0.0066 (8)	0.0073 (8)	−0.0020 (9)
C3	0.0219 (9)	0.0189 (10)	0.0217 (11)	0.0073 (8)	0.0033 (8)	−0.0019 (9)
C4	0.0289 (10)	0.0197 (10)	0.0230 (12)	0.0050 (8)	0.0097 (8)	0.0002 (10)
C5	0.0269 (10)	0.0238 (11)	0.0327 (13)	0.0090 (9)	0.0040 (9)	−0.0004 (10)
C6	0.0264 (10)	0.0198 (10)	0.0282 (12)	0.0033 (8)	0.0053 (8)	−0.0004 (10)
C7	0.0397 (13)	0.0296 (12)	0.0275 (13)	0.0038 (10)	0.0021 (10)	0.0015 (11)
C8	0.0561 (16)	0.0282 (13)	0.0248 (13)	0.0053 (11)	0.0078 (11)	−0.0054 (11)
C9	0.0522 (15)	0.0258 (12)	0.0325 (14)	0.0127 (11)	0.0153 (12)	−0.0040 (12)
C10	0.0366 (12)	0.0235 (11)	0.0333 (14)	0.0117 (10)	0.0092 (10)	0.0011 (11)
C11	0.0259 (10)	0.0195 (10)	0.0236 (12)	0.0035 (8)	0.0074 (8)	−0.0003 (9)
C12	0.0478 (15)	0.0276 (13)	0.0449 (16)	0.0002 (11)	0.0088 (12)	0.0095 (12)

### Geometric parameters (Å, º)

**Table:**

Cl1—C2	1.730 (2)	C5—C6	1.508 (3)
O1—C1	1.255 (3)	C5—H5	1.0000
O2—C3	1.251 (2)	C6—C11	1.384 (3)
O3—C5	1.413 (3)	C6—C7	1.388 (4)
O3—C12	1.422 (3)	C7—C8	1.379 (3)
N1—C4	1.319 (2)	C7—H7	0.9500
N1—C11	1.399 (3)	C8—C9	1.389 (4)
N1—H1	0.95 (3)	C8—H8	0.9500
N2—C4	1.305 (3)	C9—C10	1.374 (4)
N2—C5	1.457 (3)	C9—H9	0.9500
N2—H2	0.90 (3)	C10—C11	1.398 (3)
C1—C2	1.401 (3)	C10—H10	0.9500
C1—C3^i^	1.537 (3)	C12—H12A	0.9800
C2—C3	1.405 (3)	C12—H12B	0.9800
C3—C1^i^	1.537 (3)	C12—H12C	0.9800
C4—H4	0.9500		
			
C5—O3—C12	115.09 (18)	C11—C6—C7	118.93 (19)
C4—N1—C11	120.69 (19)	C11—C6—C5	121.3 (2)
C4—N1—H1	120.9 (17)	C7—C6—C5	119.7 (2)
C11—N1—H1	118.0 (17)	C8—C7—C6	120.7 (2)
C4—N2—C5	124.17 (18)	C8—C7—H7	119.7
C4—N2—H2	114.4 (19)	C6—C7—H7	119.7
C5—N2—H2	121.4 (19)	C7—C8—C9	119.7 (2)
O1—C1—C2	124.72 (19)	C7—C8—H8	120.2
O1—C1—C3^i^	116.54 (16)	C9—C8—H8	120.2
C2—C1—C3^i^	118.73 (18)	C10—C9—C8	120.8 (2)
C1—C2—C3	123.38 (19)	C10—C9—H9	119.6
C1—C2—Cl1	118.46 (16)	C8—C9—H9	119.6
C3—C2—Cl1	118.15 (14)	C9—C10—C11	118.9 (2)
O2—C3—C2	126.28 (19)	C9—C10—H10	120.6
O2—C3—C1^i^	115.86 (18)	C11—C10—H10	120.6
C2—C3—C1^i^	117.85 (16)	C6—C11—C10	121.0 (2)
N2—C4—N1	123.2 (2)	C6—C11—N1	119.03 (18)
N2—C4—H4	118.4	C10—C11—N1	120.0 (2)
N1—C4—H4	118.4	O3—C12—H12A	109.5
O3—C5—N2	110.61 (19)	O3—C12—H12B	109.5
O3—C5—C6	113.51 (18)	H12A—C12—H12B	109.5
N2—C5—C6	110.36 (18)	O3—C12—H12C	109.5
O3—C5—H5	107.4	H12A—C12—H12C	109.5
N2—C5—H5	107.4	H12B—C12—H12C	109.5
C6—C5—H5	107.4		
			
O1—C1—C2—C3	178.1 (2)	O3—C5—C6—C7	−63.7 (3)
C3^i^—C1—C2—C3	−2.3 (3)	N2—C5—C6—C7	171.5 (2)
O1—C1—C2—Cl1	−1.1 (3)	C11—C6—C7—C8	−1.3 (4)
C3^i^—C1—C2—Cl1	178.50 (14)	C5—C6—C7—C8	176.3 (2)
C1—C2—C3—O2	−178.8 (2)	C6—C7—C8—C9	0.3 (4)
Cl1—C2—C3—O2	0.4 (3)	C7—C8—C9—C10	0.6 (4)
C1—C2—C3—C1^i^	2.3 (3)	C8—C9—C10—C11	−0.5 (4)
Cl1—C2—C3—C1^i^	−178.52 (14)	C7—C6—C11—C10	1.4 (3)
C5—N2—C4—N1	−5.1 (3)	C5—C6—C11—C10	−176.1 (2)
C11—N1—C4—N2	−4.4 (3)	C7—C6—C11—N1	−178.9 (2)
C12—O3—C5—N2	64.7 (2)	C5—C6—C11—N1	3.6 (3)
C12—O3—C5—C6	−60.0 (3)	C9—C10—C11—C6	−0.5 (3)
C4—N2—C5—O3	−114.4 (2)	C9—C10—C11—N1	179.8 (2)
C4—N2—C5—C6	12.1 (3)	C4—N1—C11—C6	4.8 (3)
O3—C5—C6—C11	113.8 (2)	C4—N1—C11—C10	−175.5 (2)
N2—C5—C6—C11	−11.0 (3)		

Symmetry code: (i) −*x*+1, −*y*, −*z*+1.

### Hydrogen-bond geometry (Å, º)

**Table:**

*D*—H···*A*	*D*—H	H···*A*	*D*···*A*	*D*—H···*A*
N1—H1···O1	0.95 (3)	1.79 (3)	2.706 (2)	160 (3)
N1—H1···O2^i^	0.95 (3)	2.56 (3)	3.229 (3)	127 (2)
N2—H2···O2^ii^	0.90 (3)	1.87 (3)	2.762 (3)	171 (3)
C4—H4···O1^iii^	0.95	2.34	3.214 (3)	152
C10—H10···O1	0.95	2.52	3.230 (3)	131

Symmetry codes: (i) −*x*+1, −*y*, −*z*+1; (ii) *x*−1, *y*+1, *z*; (iii) −*x*+1, −*y*+1, −*z*+1.
